# Outcomes of endoscopic pyloric stenting in malignant gastric outlet obstruction: a retrospective study

**DOI:** 10.1186/1756-0500-6-280

**Published:** 2013-07-19

**Authors:** Hala Mansoor, Muhammed Aasim Yusuf

**Affiliations:** 1 Department of Internal Medicine, Shaukat Khanum Memorial Cancer Hospital and Research Centre, 7/A Block R-3 MA Johar Town, Lahore, Pakistan

**Keywords:** SEMS (self-expandable metal stents), Gastric outlet obstruction, GOOSS (gastric outlet obstruction scoring system)

## Abstract

**Background:**

Up to 30% of patients with pancreatic cancer and more than 50% of patients with gastric cancer already have incurable disease, with distressing symptoms of gastric outlet obstruction at the time of presentation which require effective palliation. We decided to test the clinical outcomes of endoscopic stent placement in malignant gastric outlet obstruction.

**Methods:**

In a retrospective single institution-based study, the charts of patients who had self-expandable metal stents placed to alleviate malignant gastric outlet obstruction were reviewed. Charts were reviewed to assess improvement in oral intake according to the Gastric Outlet Obstruction Scoring System (GOOSS), and in order to also evaluate technical success and complications of the procedure.

**Results:**

69 patients with successful stent placement were retrospectively evaluated. Within 7 and 28 days after stent placement respectively, 85.5% and 80% benefited from stent insertion, with an increase in the GOOSS score of > 1. Resumption of soft or low residue diet (GOOSS 2-3) was achieved in 53.6% at day 7 and in 62% of patients at day 28, respectively. Of the patients achieving a GOOSS score of 2-3, 17.3% remained on a soft or low residue diet at 24 weeks or at last follow up, while 46% died. Stent related adverse events occurred in 10 patients (14%), including stent blockade in 7 and stent migration in 3 patients.

**Conclusion:**

Endoscopic enteral stenting promptly increases oral intake in the majority of patients with malignant gastric outlet obstruction and is a safe procedure with a low rate of serious complications.

## Background

Malignant gastric outlet obstruction occurs in advanced gastric, duodenal and pancreatobiliary malignancies. It leads to intolerance of oral intake by causing vomiting, resulting in weight loss and impaired quality of life. Recurrent vomiting also puts patients at risk of aspiration pneumonia [1-3]. Advances in chemotherapeutic agents have improved survival in many of the malignancies cited above [4]. A number of the newer anti-cancer agents used for treating advanced gastric and pancreato-biliary cancers are oral, such as capecitabine and erlotinib, so maintaining oral intake is also helpful in providing treatment for many of these tumors [5-11].

Traditionally, the standard treatment of malignant gastric outlet obstruction has been surgical gastro-jejunostomy. The advent of endoscopically-placed self-expandable metallic stents has now widely changed practice, both in the palliative setting in advanced malignancies with gastric outlet obstruction as well as in the curative setting, where it is important to relieve gastric outlet obstruction so as to maintain nutrition during neo-adjuvant chemotherapy [12,13]. In patients with limited life expectancy, metallic stent insertion serves as an excellent palliative measure for several reasons. It can be carried out as an outpatient procedure, is cheap compared to surgery, has high clinical success rates, has low incidence of delayed gastric emptying, reduced morbidity and allows rapid resumption of oral feeding when compared with surgical gastro-jejunostomy [14-17].

The aim of this study was to retrospectively analyze the data of our hospital patients to determine the outcome of stent placement at our institution. The main emphasis of the study was to assess technical success rates and complications of the procedure, as well as to evaluate improvement in oral intake after stent placement. This was evaluated by assessing oral intake using the four-point Gastric Outlet Obstruction Scoring System (GOOSS) by retrospective data collection. Any procedure-related adverse events were also studied to assess the safety of the procedure.

## Methods

### Patients

This retrospective study was conducted at Shaukat Khanum Memorial Cancer Hospital & Research Centre, Lahore, Pakistan, in accordance with the principles of the Helsinki Declaration and after obtaining approval from the Shaukat Khanum Memorial Cancer Hospital & Research Centre Institutional Review Board. The data of 69 consecutive patients (37 males, age range 23-81 years; mean 52.8 years) who had documented gastric outlet obstruction and who underwent endoscopic stenting from August 2008 till January 2012 was reviewed retrospectively. All patients had symptomatic gastric outlet obstruction that was characterized by vomiting and reduced oral intake, and confirmed at endoscopy or radiologically. Endoscopic pyloric stenting was the primary treatment option to treat gastric outlet obstruction, in accordance with decisions made in multidisciplinary meetings. Palliative gastro-jejunostomy was reserved as a second line measure in the event of failure of endoscopic stenting or for stent blockade not amenable to endoscopic re-stenting. Mildly symptomatic patients, in whom an adult endoscope could be passed through the pylorus with ease, were not stented.

### Procedure of stent placement

Initial upper GI endoscopy was performed to assess the nature and site of obstruction. A biliary catheter and a floppy guide-wire were used to negotiate the stricture and contrast was injected under fluoroscopic control to assess the length and extent of the stricture. The stent delivery assembly was passed through the working channel of a therapeutic gastroscope, over the guide-wire, and deployed under direct vision, with adjunct fluoroscopic control, in a standard manner. Patients usually resumed oral intake of liquids within 6 hours of stent placement. Diet was gradually advanced to a liquid/pureed diet over 24 hours, and to a soft diet thereafter, as tolerated.

### Definitions

The outcome of SEMS placement was evaluated according to the following parameters (1) technical success, (2) clinical success, (3) complications. Technical success was defined as correct placement of a stent in an appropriate position, as confirmed by fluoroscopy. Symptoms consistent with gastric outlet obstruction were defined as nausea, vomiting and early satiety. The four point Gastric Outlet Obstruction Scoring System (GOOSS) was retrospectively used to assess the utility of stent insertion in relieving obstruction : (0) no oral intake; (1) exclusively liquid diet; (2) soft solids only; (3) low residue or full diet. We calculated the GOOSS score retrospectively for all patients, at 7 days, 28 days and 24 weeks after stent placement. Clinical success was defined as improvement in the GOOSS score after stenting.

Primary stent failure was defined as failure to resume any oral intake after stent placement. Complications were classed as either procedure-related, if they occurred during endoscopy, or device-related if they occurred later, and included stent blockade, migration, cholangitis, duodenal perforation and intestinal hemorrhage.

### Follow up

The patient’s charts were reviewed retrospectively to assess clinical outcome and complications. Data was obtained from the hospital records including inpatient admission notes, clinic and emergency room visit notes, endoscopy reports, radiology reports and from patients or their families by telephonic enquiry. A follow up endoscopy or barium study was performed to evaluate stent malfunction only in patients with recurrent symptoms suggestive of obstruction.

### Statistical analysis

The degree of oral intake before and after stent placement was analyzed by SPSS version 19.

## Results

### Patient characteristics

77 stent insertion procedures were attempted in 69 patients (37 males, age range 23-81 years; mean 52.8 years). 7 procedures were performed in patients with a blocked stent. 5 of these 7 patients had a successful stent insertion through a previously inserted stent to treat stent blockade from tumor ingrowth or overgrowth. One patient needed re-stenting to manage stent migration. 44 patients had gastric carcinoma, 11 had pancreatic and periampullary tumors, 5 had carcinoma of the gall bladder and 3 had ovarian cancer while there were 2 patients with diffuse large B cell lymphoma and one patient each with duodenal, breast and rectal cancers, and one patient with a retroperitoneal liposarcoma. The site of obstruction was antro-pylorus in 45, duodenum in 20, pylorus and duodenum in 3 and at the site of a previous gastrojejunostomy in 1 patient.

### Technical and clinical success

The technical success rate was 97%. At baseline, 68% of patients had a GOOSS score of 0, while 30.4% had a score of 1 (Table 1). By day 7, the GOOSS score had increased > 1 in 85.5% patients, out of which the score had improved to 2-3 in 53.6% (Table 2), a proportion that increased to 62% by day 28 (Table 3). However, by 24 weeks, 46% of patients had died, 8.7% had a score of only 1, while 18.8% had a score of 2–3 (Table 4). Four patients underwent surgical resection of gastric tumours following stent insertion. These were all patients who initially received neo-adjuvant chemotherapy and in whom the tumour was judged to be resectable at 24 weeks. 9 patients were lost to follow up and could not be contacted by telephone.

**Table 1:** Baseline GOOSS

**GOOSS**	**Frequency**	**Percent**
0	47	68.1
1	21	30.4
2	1	1.4
Total	69	100.0

*GOOSS* Gastric outlet obstruction scoring system.

**Table 2:** Day 7 GOOSS

**GOOSS**	**Frequency**	**Percent**
0	2	2.9
1	22	31.9
2	29	42.0
3	8	11.6

*GOOSS* Gastric outlet obstruction scoring system.

**Table 3:** GOOSS day 28

**GOOSS**	**Frequency**	**Percent**
0	1	1.4
1	12	17.4
2	36	52.2
3	7	10.1

*GOOSS* Gastric outlet obstruction scoring system.

**Table 4:** GOOSS week 24

**GOOSS**	**Frequency**	**Percent**
0	3	4.3
1	6	8.7
2	10	14.5
3	3	4.3

*GOOSS* Gastric outlet obstruction scoring system.

A subgroup analysis was performed to assess the effects of chemotherapy on stent patency. Out of 56 patients who could be evaluated by day 28, 25 patients had received chemotherapy. 14 of these 25 patients had a GOOSS score of 0 at baseline and 11 had a score of 1. At day 28, 6 patients had a GOOSS score of 1 and 19 had a score of 2-3. Out of 31 patients who did not receive chemotherapy, 25 patients had a GOOSS score of 0 at baseline and 6 had a score of 1. At day 28, 1 patient had a GOOSS score of 0, 6 had a score of 1 while 24 had a score of 2-3 (p value = 0.139).

By 24 weeks, only 22 patients were evaluable. 32 had died, 6 had undergone surgery and 9 were lost to follow-up. Of these 22 patients, 11 had received chemotherapy and a similar number had not. Of the 11 patients who received chemotherapy, the baseline GOOSS score was 0 in 6 patients and 1 in 5 patients. By 24 weeks, the scores were 0 in 1 patient, 1 in 1 patient and 2-3 in nine patients. Of the 11 patients who did not receive chemotherapy, baseline GOOSS scores were 0 in 8 patients and 1 in 3 patients. At 24 weeks, these were 0 in 2 patients, 1 in 5 patients and 2-3 in 4 patients (p value = 0.001).

### Complications

Ten patients (14%) experienced device-related adverse effects. Seven patients had stent blockade and three had stent migration. Of the patients who had stent blockade, this occurred within 28 days of insertion in one patient, between one and six months in three patients and between eight to twelve months post-stenting in three. Stent blockade was usually found to be secondary to tumor ingrowth/overgrowth and new stents were successfully inserted through the previously placed stents in five of these seven patients. The remaining two patients underwent palliative gastro-jejunostomy. Three patients had stent migration between 7-42 days post-stenting. One patient required repositioning of the stent, in another a new stent was inserted while the third patient was managed conservatively and allowed home without further intervention in view of his very short life-expectancy at that time. No other serious adverse events occurred.

## Discussion

Malignant gastric outlet obstruction greatly impairs the quality of life by causing nausea, vomiting, abdominal distension, dehydration, electrolyte imbalance, starvation and weight loss [1-3,18]. Up to 30% of patients with pancreatic and more than 50% of patients with gastric cancer already have incurable disease, with distressing symptoms of gastric outlet obstruction at the time of presentation, and require effective palliation. The principal objective of palliative treatment in this situation is the resolution of obstructive symptoms and resumption of oral intake [19-21].

Insertion of SEMS has emerged as a safe and effective means of palliation, avoiding the added risks of anaesthesia and surgery. Trials and meta-analyses have shown that SEMS placement is superior to surgical gastro-enterostomy, as evidenced by earlier resumption of oral intake and shorter hospital stay [12,13,22,23]. SEMS placement can be offered as first-line treatment of gastric outlet obstruction in most patients, or as a second-line therapy where surgery or other oncologic treatments have failed to provide relief [17].

The GOO scoring system was first introduced in 2002, by Adler and Baron, to allow clinical grading of gastric outlet obstruction before and after treatment. The scale is now widely used and a number of studies have been carried out using this scale, in order to evaluate oral intake before and after stent placement. A number of these have shown a favourable outcome with SEMS placement [18,24,25]. In our study, improvement in the GOOSS score was one of the parameters studied. Within 7 days of stent placement, 85.5% of patients had attained a GOOSS score increase of > 1, and 53.6% had an improvement in GOOSS score to 2-3, implying resumption of a soft or low residue diet. Of the patients attaining an initial GOOSS score increase of > 1 and an absolute score of 2-3, respectively 80% and 62% maintained these improvements for 28 days, despite progressive disease in many patients.

Our findings are largely consistent with previous studies. In a European multicenter study, van Hooft et al. carried out a retrospective data analysis of 62 patients in whom WallFlex enteral stents were inserted. All 56 evaluable patients had resumed some form of oral intake by the end of week 1, consisting of liquids in 7 patients, soft food in 17 and a low residue or full diet in 32 patients [26]. In a retrospective study of 95 patients with gastric cancer, Cho et al. reported a technical success rate of 98% for stent placement, and a clinical success rate, defined as improvement in oral intake and obstructive symptoms 1-3 days after stent placement, of 87%. The GOOSS score was used to assess improvement in oral intake. Recurrent symptoms of obstruction were observed in 25/81 cases (30.8%), due to tumor ingrowth in 23 and overgrowth in 2 patients. Mean survival was 129 days. Serious complications occurred in two patients and were bowel perforation in one patient and aspiration pneumonia in another [27].

In the prospective DUOFLEX study, the efficacy and safety of the Wallflex stent in gastric outlet obstruction was evaluated in 51 patients. Technical success was achieved in 98% and clinical success was seen in 84% of patients respectively, with 307 days of median stent patency (75% functional at 135 days and 25% at 470 days) and median survival of 62 days (75% alive at 35 days and 25% at 156 days). This large difference between median stent patency and median survival suggests that enteral stenting adequately relieved GOO in the majority of patients until death. 7 patients had stent dysfunction; migration in 1 and tumor ingrowth or overgrowth in 6. The GOOSS score improved significantly (p < 0.001) and the World Health Organization performance score also improved, when the pre-stenting score was compared with the mean score till death (p = 0.002). However, the global quality of life did not improve, and the body mass index decreased over time (p < .001), suggesting that in palliation of patients with advanced cancer causing GOO, attempts should be made not only to improve the passage of food, but to also consider other factors that might impair the quality of life including pain and psychological factors [28].

In another prospective study of 37 patients performed at three referral hospitals in Japan, the technical success rate was reported as 97% and the clinical success rate was 94.4%. However, the complication rate was relatively high at 16.2%, with two cases of primary stent dysfunction, 1 perforation, 1 gastrointestinal bleed, 1 blocked stent and one case of biliary stent dysfunction. The follow-up period was also relatively short, with a median follow-up period of 68 days [29]. In a retrospective Korean study of 82 patients, Morikawa et al. reported a technical success rate of 93.9%, with mean GOOSS score improving from 0.56 before stenting to 1.92 (p < 0.001) after stenting. Complications were seen in 12 (14.6%) patients, with 10/12 developing stent blockade due to tumor ingrowth and one patient each with stent migration and perforation. Median survival post stenting was 52 days (range 6-445 days) [30].

In a larger prospective study of 213 patients, the role of chemotherapy in maintaining stent patency was also studied. Technical and clinical success rates were comparable with other studies, at 94% each. The mean and median stent patency periods were 324 and 270 days, respectively. The mean and median survival recorded were 159 (CI 116-203) and 99 (CI 78-121) days, respectively. Maintenance of stent patency was significantly higher in those who received chemotherapy after SEMS insertion (p < 0.001), although stent migration rates were also increased [31]. In another study of SEMS placement in patients with biliary tract malignancy causing duodenal obstruction, stent placement was successful in all 17 patients and all patients were able to tolerate soft or solid food by 4 weeks. There were no procedure-related deaths [32].

In comparing our outcomes with the literature, we found that our technical and clinical success rates were comparable to most studies [26-28,30], although the study by Kim et al. had better clinical outcomes in terms of longer stent patency related to the effect of chemotherapy [31]. Subgroup analysis of our patients who received chemotherapy did not show a significant difference in GOOSS score by day 28 post-stenting when compared with those who had not received chemotherapy. However, a better clinical outcome, in terms of better oral intake and less obstructive symptoms by 24 weeks post stenting was seen (82% with a score of 2-3 in the chemotherapy group vs 36% with a score of 2-3 in those who did not receive chemotherapy). Clearly, however, the number of patients was too small to establish any clinical significance. Although serious complications associated with SEMS insertion have been reported, such as gastrointestinal hemorrhage, sepsis, cholangitis, bowel perforation and aspiration pneumonia [18,27,29,30], the incidence of such complications is low, with most complications being non-fatal [27-30,32]. In our study, there were no incidents of stent-related bowel perforation, aspiration pneumonia, cholangitis or sepsis. The median survival time in malignancies with gastric outlet obstruction is generally short, ranging from 49-99 days. In our study, 46% of patients had died by 24 weeks, but, as already discussed, it has been shown that the lower morbidity and earlier resumption of oral intake in these patients does lead to an improved quality of life when compared with those treated surgically.

## Conclusions

From our experience, it seems clear that endoscopic stent placement ought to be considered a first-line measure to relieve gastric outlet obstruction in this population with a limited life expectancy. Further controlled studies are required to assess the role of chemotherapy, radiotherapy and the effect of the primary malignancy on stent patency.

## Abbreviations

GOOSS: Gastric outlet obstruction scoring system; SEMS: Self expandable metal stents; GI: Gastrointestinal.

## Competing interests

There were no financial or non–financial interests involved in this study.

## Authors’ contributions

HM participated in the design of the study, acquired the data, performed the statistical analysis and helped to draft the manuscript. MAY performed the endoscopic procedures, was involved in drafting the manuscript, revised it critically and gave final approval of the version to be published.Both authors read and approved the final manuscript.

## Authors’ information

HM: MRCP UK, FCPS Medicine, is a Gastroenterology Fellow at Shaukat Khanum Memorial Cancer Hospital & Research Centre Lahore, Pakistan. MAY: FRCP Edin, is a Consultant Gastroenterologist at Shaukat Khanum Memorial Cancer Hospital & Research Centre, Lahore, Pakistan.
